# Electrospun Antimicrobial Films of Poly(3-hydroxybutyrate-*co*-3-hydroxyvalerate) Containing Eugenol Essential Oil Encapsulated in Mesoporous Silica Nanoparticles

**DOI:** 10.3390/nano9020227

**Published:** 2019-02-08

**Authors:** Beatriz Melendez-Rodriguez, Kelly J. Figueroa-Lopez, Andrea Bernardos, Ramón Martínez-Máñez, Luis Cabedo, Sergio Torres-Giner, Jose M. Lagaron

**Affiliations:** 1 Novel Materials and Nanotechnology Group, Institute of Agrochemistry and Food Technology (IATA), Spanish Council for Scientific Research (CSIC), Calle Catedrático Agustín Escardino Benlloch 7, 46980 Paterna, Spain; beatriz.melendez@iata.csic.es (B.M.-R.); kjfigueroal@iata.csic.es (K.J.F.-L.); storresginer@iata.csic.es (S.T.-G.); 2 Instituto Interuniversitario de Investigación de Reconocimiento Molecular y Desarrollo Tecnológico (IDM), Universitat Politècnica de València (UPV), Universitat de València (UV), camí de Vera s/n, 46022 Valencia, Spain; anberba@upv.es (A.B.); rmaez@qim.upv.es (R.M.-M.); 3 CIBER de Bioingeniería, Biomateriales y Nanomedicina (CIBER-BBN), Camino de Vera s/n, 46022 Valencia, Spain; 4 Unidad Mixta de Investigación en Nanomedicina y Sensores, Universitat Politècnica de València (UPV), Instituto de Investigación Sanitaria La Fe, 46026 Valencia, Spain; 5 Unidad Mixta UPV-CIPF de Investigación en Mecanismos de Enfermedades y Nanomedicina, Universitat Politècnica de València (UPV), Centro de Investigación Príncipe Felipe, 46012 Valencia, Spain; 6 Polymers and Advanced Materials Group (PIMA), Universitat Jaume I, 12071 Castellón, Spain; lcabedo@uji.es

**Keywords:** PHBV, MCM-41, eugenol, antimicrobial properties, active packaging

## Abstract

The main goal of this study was to develop poly(3-hydroxybutyrate-*co*-3-hydroxyvalerate) (PHBV) films with long-term antimicrobial capacity of interest in food packaging applications. To this end, eugenol was first highly efficiently encapsulated at 50 wt.-% in the pores of mesoporous silica nanoparticles by vapor adsorption. The eugenol-containing nanoparticles were then loaded in the 2.5–20 wt.-% range into PHBV by electrospinning and the resultant electrospun composite fibers were annealed at 155 °C to produce continuous films. The characterization showed that the PHBV films filled with mesoporous silica nanoparticles containing eugenol present sufficient thermal resistance and enhanced mechanical strength and barrier performance to water vapor and limonene. The antimicrobial activity of the films was also evaluated against foodborne bacteria for 15 days in open vs. closed conditions in order to simulate real packaging conditions. The electrospun PHBV films with loadings above 10 wt.-% of mesoporous silica nanoparticles containing eugenol successfully inhibited the bacterial growth, whereas the active films stored in hermetically closed systems increased their antimicrobial activity after 15 days due to the volatile portion accumulated in the system’s headspace and the sustained release capacity of the films. The resultant biopolymer films are, therefore, potential candidates to be applied in active food packaging applications to provide shelf life extension and food safety.

## 1. Introduction

Polyhydroxyalkanoates (PHAs) currently represent one of the most important alternative to petroleum-based materials in the frame of the Circular Economy [1]. PHAs, which are synthesized by a wide range of microorganisms as carbon storage material, are thermoplastic materials, biodegradable, and present similar physical properties to other plastics, e.g., polypropylene (PP) and polystyrene (PS), such as high mechanical strength and water resistance [2]. PHAs have been prompted as potential packaging applications due to their biocompatibility and physical properties [3]. However, the PHA production currently associates a high cost due to the carbon sources of the raw materials, i.e., low yield and productivity, and the down-stream process [4]. The synthesis of PHA through fermentation from industrial by-products and waste, particularly the use of mixed microbial cultures, is nowadays seen as an option to reduce the production costs [5].

Among PHAs, the most widely studied and easiest-to-produce member of this family is poly(3-hydroxybutyrate) (PHB). This isotactic homopolyester presents a relatively high melting temperature (T_m_) and good stiffness due to its high crystallinity (>50%). However, the use of PHB has been limited due to several drawbacks, particularly its poor impact-strength resistance and low thermal stability. To overcome these shortcomings, the use of its copolymers, such as those made with 3-hydroxyvalerate (3HV) or 4-hydroxybutyrate (4HB) to produce poly(3-hydroxybutyrate-*co*-3-hydroxyvalerate) (PHBV) and poly(3-hydroxybutyrate-*co*-4-hydroxybutyrate) (P(3HB-*co*-4HB)), can improve these limitations and widen its processing window [6,7]. In particular, PHBV is a potential candidate to be applied for packaging of films, blow-molded bottles, paper coatings, etc. [8]. To this end, different studies have explored the use of PHBV due to its potential as a sustainable packaging material [9,10]. For instance, PHBVs have been applied in the form of films, fibers, and foams for everyday articles such as shampoo bottles and plastic beverage bottles due to its renewability, biodegradability, and high water vapor barrier [11]. In addition, the incorporation of antimicrobial and/or antioxidant substances into a PHA-based packaging material can result in high interest to improve both protection and shelf life of foodstuffs during the storage period [12,13,14].

Electrospinning is an innovative technology to generate ultrathin fibrous mats made of a wide range of polymer and biopolymer materials with fiber diameters ranging from several nanometers to a few microns [15]. Electrospun ultrathin fibers have prompted their use in a wide range of industrial sectors, including packaging applications [16,17]. This technique is highly suitable for the encapsulation and/or sustained delivery of active and bioactive substances at the nanoscale level due to both the high surface-to-volume ratios of the electrospun fibers and the high porosity of their mats [18,19]. In particular, electrospinning is interesting for the development of antimicrobial materials by either the use of inherently antimicrobial polymers or the nanoencapsulation of biocide substances [20]. As a result, within the frame of active packaging, different recent studies have reported the encapsulation of metal nanoparticles (MNPs) in electrospun matrices. For instance, poly(vinyl alcohol) (PVOH) and poly(N-isopropylacrylamide) (PNIPAAm) membranes containing silver nanoparticles (AgNPs) immobilized onto cellulose nanowhiskers (CNWs) presented antimicrobial activity against several Gram-negative (G-) and Gram-positive (G+) bacteria [21]. In another study, polyvinylpyrrolidone (PVP)/poly(ε-caprolactone) (PCL) nanofibers functionalized with zinc oxide nanoparticles (ZnONPs) and AgNPs, also prepared by electrospinning, showed a high antibacterial activity against *Staphylococcus aureus* (*S. aureus*) and *Escherichia coli* (*E. coli*) [22]. Similarly, electrospun chitosan/poly(ethylene oxide) (PEO) membranes containing AgNPs presented antimicrobial effect against *E. coli* [23]. Recently developed electrospun PHA materials containing AgNPs [24] and copper oxide nanoparticles (CuONPs) [25] have been also able to considerably reduce bacterial growth at very low contents. These novel NPs-containing electrospun materials offer significant potential as new antimicrobial coatings or interlayers, that is, internal layers in a multilayer system, for application in the design of active food packaging structures.

Natural antimicrobials, such as essential oils (EOs), are currently regarded as an alternative to synthetic preservatives of food because they are considered as Generally Recognized As Safe (GRAS) substances, being acceptable to consumers [26] and having the capacity to exert a multitude of biological effects [27]. For instance, eugenol, which has potential antimicrobial and antioxidant actions, has been effectively applied against foodborne pathogens [28,29]. However, EOs are frequently unstable and can be easily degraded in stressful situations such as in the presence of oxygen, temperature and light, so that they can lose their antimicrobial activity [30]. To avoid this issue, encapsulation is considered a good way to protect and preserve the effectiveness of active and bioactive substances [31]. In this sense, silica mesoporous supports (SMPSs) [32] show a great deal of potential for the storage and release of active substances [33,34]. In particular, the typical sizes of SMPSs range from microns to nanometers, presenting tailor-made pores of around 2–10 nm [35]. The particular morphology of SMPSs renders a very large specific surface area, up to 1200 m^2^/g and, then, an enhanced loading capacity for the encapsulation and release of natural antimicrobials [36]. Within SMPSs, Mobil Composition of Matter (MCM), including both MCM-41 and MCM-48, are among of the most popular mesoporous molecular sieves in which their pore diameter can be nicely controlled by adjusting their synthesis conditions and/or by employing surfactants with different chain lengths in their preparation [37]. Silica mesoporous materials are thus able to encapsulate organic molecules, forming host–guest complexes with volatile molecules (e.g., EOs) to efficiently control their volatility and reactivity. So far, many studies have employed MCM to encapsulate active substances with positive results in different applications, for instance, caprylic acid against foodborne pathogens [38], EOs as antifungal [36,39,40] and antimicrobial systems [41], and poplar-type propolis in drug delivery platforms [42]. In particular, the antimicrobial and antifungal effect of the EOs-functionalized supports improved compared to the free compounds due to the EOs encapsulated inside MCM released in a controlled manner [39,40,41]. These previous results suggest that the immobilization of EOs onto silica supports can represent a novel strategy to develop a new generation of long-term antimicrobial systems that may not only enhance the antimicrobial activity of EOs, but also mask their characteristic odor/taste for food-related applications.

In this study, it is initially reported the preparation of nanometric MCM-41 particles loaded with eugenol, a phenylpropene and an allyl chain-substituted guaiacol that is primarily extracted from cinnamon, bay leaf, nutmeg, basil, and clove [43]. The resultant MCM-41 particles containing eugenol were thereafter incorporated, for the first time, into PHBV by electrospinning. The generated electrospun composite fibers were thermally post-treated to produce films that were characterized in terms of their morphology, thermal, mechanical, and barrier properties. Finally, the antimicrobial performance against foodborne bacteria was also determined. In a packaging context, the active tests were carried out as a function of time in open vs. close conditions in order to simulate potential real conditions.

## 2. Materials and Methods

### 2.1. Materials

Commercial PHBV was ENMAT^TM^ Y1000P, produced by Tianan Biologic Materials (Ningbo, China) and delivered in the form of pellets by NaturePlast (Ifs, France). According to the manufacturer, this biopolymer resin presents a density of 1.23 g/cm³ and a melt flow index (MFI) of 5–10 g/10 min (190 °C, 2.16 kg). The 3HV fraction in the copolyester is 2–3 mol.-%.

Eugenol, with 99% purity, tetraethyl orthosilicate (TEOS), n-cetyltrimethylammonium bromide (CTAB), sodium hydroxide (NaOH), 2,2,2-trifluoroethanol (TFE), ≥99% purity, and d-limonene, with 98% purity, were all purchased from Sigma Aldrich S.A. (Madrid, Spain).

### 2.2. Synthesis and Complexation of MCM-41

#### 2.2.1. Synthesis of MCM-41

The MCM-41 type mesoporous particles were synthesized using the following procedure [44]: 2 g of CTAB, 5.48 mmol, was first dissolved in 960 mL of deionized water. Then, 7.00 mL of NaOH, 2 M, was added to the CTAB solution, followed by adjusting the solution temperature to 95 °C. Later, 10 mL of TEOS, 5.14·10^−2^ mol, was added dropwise to the surfactant solution. The mixture was allowed to stir for 3 h to produce a white precipitate. The solid product was centrifuged and washed several times with deionized water and ethanol and, thereafter, dried at 60 °C to obtain solid MCM particles. Lastly, to prepare the final porous material, i.e., the MCM-41 type particles, the as-synthetized MCM particles were calcined at 550 °C using air atmosphere for 5 h so that their template phase was removed.

#### 2.2.2. Eugenol Complexation on MCM-41

Silica loading with eugenol was achieved via vapor adsorption by mixing 100 mg of eugenol with 100 mg of the MCM-41 type particles in a tightly closed vial [36]. The mixture was incubated in an oven at 40 °C for 24 h while being continuously shaken. The amount of eugenol loaded in the MCM-41 type support was determined by monitoring the sample weight increase before and after the loading process. Approximately 500 mg/g of the final weight corresponded to eugenol.

### 2.3. Electrospinning Process

Prior to electrospinning, different PHBV solutions were prepared by dissolving the biopolymer at 10 wt.-% in TFE. Then, the MCM-41 type particles, with and without eugenol, were added to the PHBV solutions at 2.5, 5, 7.5, 10, 15, and 20 wt.-%. A neat PHBV solution without MCM-41 type particles was also prepared as a control sample. All PHBV solutions were processed by electrospinning using a high-throughput Fluidnatek^®^ LE-500 pilot-plant device with temperature and relative humidity (RH) control manufactured by Bioinicia S.L. (Valencia, Spain). The equipment was operated in the lab mode using a motorized single needle injector, scanning vertically towards a metallic fixed collector. The conditions were set at a flow-rate of 6 mL/h, 20 kV of voltage, and 15 cm of needle-to-collector distance. Each solution was electrospun for 2 h at 25 °C and 40% RH. The collected mats were stored in darkness at room temperature in a desiccator at 0% RH for one week before physical characterization.

### 2.4. Film Preparation

The resultant electrospun PHBV fibers mats were subjected to annealing in a 4122-model press from Carver, Inc. (Wabash, IN, USA) at 155 °C, for 5 s, without pressure. These conditions were selected based on our previous work [45]. The thermally post-processed samples had an average thickness of approximately 60 µm.

### 2.5. Characterization

#### 2.5.1. Electron Microscopy 

The morphologies of the MCM-41 type particles as well as the electrospun PHBV fibers and films were observed by scanning electron microscopy (SEM) using an S-4800 device from Hitachi (Tokyo, Japan). The samples were fixed to beveled holders using conductive double-sided adhesive tape and sputtered with a mixture of gold-palladium under vacuum prior to observation. An accelerating voltage of 10 kV was used. For the cross-section observations, the films were previously cryo-fractured by immersion in liquid nitrogen. 

Detailed morphology of the MCM-41 particles and their distribution in the PHBV fibers was further studied by transmission electron microscopy (TEM) using a JEOL 1010 from JEOL USA, Inc. (Peabody, MA, USA) using an accelerating voltage of 100 kV. The estimation of the dimensions was performed by means of the Aperture software from Apple (Cupertino, CA, USA) using a minimum of 20 SEM or TEM micrographs in their original magnification.

#### 2.5.2. Thermal Analysis

Thermal transitions were studied by differential scanning calorimetry (DSC) on a DSC-7 analyzer from PerkinElmer, Inc. (Waltham, MA, USA), equipped with a cooling accessory Intracooler 2 also from PerkinElmer, Inc. A heating program was applied from −30 °C to 190 °C, followed by a cooling program to −30 °C. The heating and cooling rates were both set at 10 °C/min under nitrogen atmosphere with a flow-rate of 20 mL/min. The typical sample weight was ~3 mg while an empty aluminum pan was used as reference. Calibration was performed using an indium sample. All tests were carried out, at least, in duplicate.

Thermogravimetric analysis (TGA) was performed in a TG-STDA model TGA/STDA851e/LF/1600 thermobalance from Mettler-Toledo, LLC (Columbus, OH, USA). The samples, with a weight of about 15 mg, were heated from 50 °C to 800 °C at a heating rate of 10 °C/min under a nitrogen atmosphere with a flow-rate of 50 mL/min.

#### 2.5.3. Mechanical Tests

Tensile tests of the PHBV films were performed according to ASTM standard method D638 using an Instron 4400 universal testing machine, equipped with a 1-kN load cell, from Instron (Norwood, MA, USA). The tests were performed, at room conditions, with 115 × 16 mm^2^ stamped dumb-bell shaped specimens using a cross-head speed of 10 mm/min. Samples were conditioned for 24 h prior to tensile assay. A minimum of six specimens was measured for each sample and the average values with standard deviation (SD) were reported.

#### 2.5.4. Permeability Tests

The water vapor permeability (WVP) of the film samples was determined using the gravimetric method ASTM E96-95 in triplicate. For this, 5 mL of distilled water was placed inside a Payne permeability cup (diameter of 3.5 cm) from Elcometer Sprl (Hermallesous-Argenteau, Belgium). The films were not in direct contact with water but exposed to 100% RH on one side and secured with silicon rings. The samples were placed within a desiccator, filled with dried silica gel, at 0% RH and 25 °C. The control samples were cups with aluminum films to estimate the solvent loss through the sealing and samples placed in cups but without permeant to compensate for mass losses due to eugenol release. The cups were weighted periodically using an analytical balance (±0.0001 g). WVP was calculated from the regression analysis of weight loss data vs. time and the weight loss was compensated by the marginal losses through the sealing and eugenol release. The permeability was obtained by multiplying the permeance by the film thickness.

Similar as described above for WVP, limonene permeability (LP) was measured placing 5 mL of d-limonene inside the Payne permeability cups. The cups containing the films were placed at the controlled room conditions of 25 °C and 40% RH. The samples were measured in triplicate and the limonene vapor permeation rate (LPRT) values were estimated from the steady-state permeation slopes and the weight loss was compensated by the comparatively marginal loss through the sealing and by the fluctuations in mass of the films due to eugenol evaporation and potential water sorption. LP was calculated taking into account the average film thickness in each case.

### 2.6. Antimicrobial Assays

The antibacterial activity of the neat eugenol, the eugenol-containing MCM-41 particles, and the electrospun films with MCM-41 with eugenol was evaluated against *S. aureus* CECT240 (ATCC 6538P) and *E. coli* CECT434 (ATCC 25922). These strains were obtained from the Spanish Type Culture Collection (CECT, Valencia, Spain) and stored in phosphate buffered saline (PBS) with 10 wt.-% tryptic soy broth (TSB, Conda Laboratories, Madrid, Spain) and 10 wt.-% glycerol at −80 °C. Previous to each study, a loopful of each bacteria was transferred to 10 mL of TSB and incubated at 37 °C for 24 h. A 100-µL aliquot from the culture was again transferred to TSB and grown at 37 °C to the mid-exponential phase of growth. An approximate count of 5 × 10^5^ colony-forming units (CFU)/mL of a culture resulted in an absorbance value of 0.20, as determined by optical density at 600 nm (UV 4000 spectrophotometer, Dinko Instruments, Barcelona, Spain). 

The minimum inhibitory concentration (MIC) and minimum bactericide concentration (MBC) of eugenol against the selected foodborne bacteria was tested following the plate micro-dilution protocol, as described in the Methods for Dilution Antimicrobial. Susceptibility Tests for Bacteria That Grow Aerobically; Approved Standard Tenth. Edition (M07-A10) by the Clinical and Laboratory Standards Institute (CLSI). For this, a 96-well plate with an alpha numeric coordination system (columns 12 and rows A-H) were used, where 10 µL of the tested samples were introduced in the wells with 90 µL of the bacteria medium. In the wells corresponding to A, B, C, E, F, and G columns different concentrations of eugenol, that is, 0.312, 0.625, 1.25, 2.5, 5, 10, 20, 40, 80, 160 µL/mL, were tested, in triplicate, from rows 1 to 10. Columns D and H were used as control of eugenol in TSB without bacteria. Row 11 was taken as positive control, that is, only TSB, and row 12 was used as negative control, that is, *S. aureus* and *E. coli* in TSB. The plates were incubated at 37 °C for 24 h. Thereafter, 10 µL of resazurin, a metabolic indicator, was added to each well and incubated again at 37 °C for 2 h. Upon obtaining the resazurin change, the wells were read through color difference. The MIC value was determined as the lowest concentration of eugenol presenting growth inhibition. 

The antimicrobial performance of the films was evaluated by using a modification of the Japanese Industrial Standard JIS Z2801 (ISO 22196:2007). A microorganism suspension of *S. aureus* and *E. coli* was applied onto the test films of PHBV/MCM-41 with eugenol and also PHBV/MCM-41, as negative control without eugenol, both sizing 2 × 2 cm^2^. After incubation for 24 h at 24 °C and at a RH of at least 95%, bacteria were recovered with PBS, 10-fold serially diluted and incubated at 37 °C for 24 h in order to quantify the number of viable bacteria by conventional plate count. The antimicrobial activity was evaluated from 1 (initial day), 8, and 15 days. The antibacterial activity was taken as the test surface reduction (R) using the equation 1:

R = [log(B/A) − log(C/A )] = log(B/C),(1)

where A is the mean of bacterial counts of the control sample immediately after inoculation, B is the mean of bacterial counts of the control sample after 24 h, and C is the mean of bacterial counts of the test sample after 24 h. Antimicrobial activity was evaluated with the following assessment: Nonsignificant (R < 0.5), slight (R ≥ 0.5 and <1), significant (R ≥ 1 and <3), and strong (R ≥ 3) [46].

## 3. Results

### 3.1. Morphology

**Figure 1:**
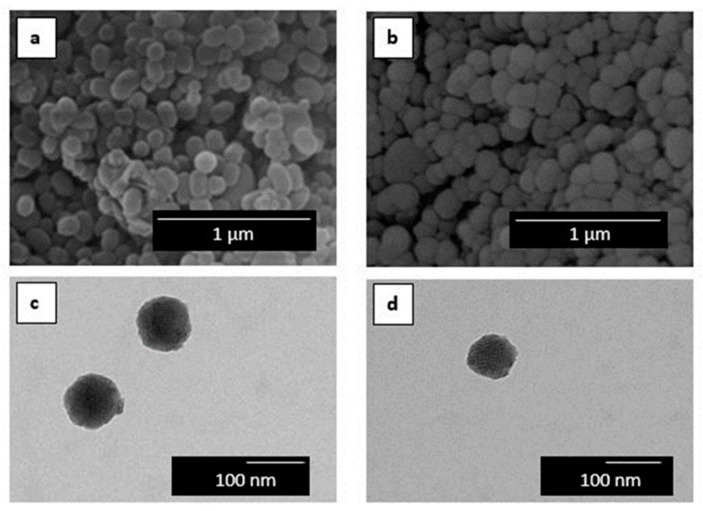
Scanning electron microscopy (SEM) images of: (**a**) Mobil Composition of Matter (MCM-41); (**b**) MCM-41 with eugenol. Scale markers of 1 µm. Transmission electron microscopy (TEM) images of: (**c**) MCM-41 and (**d**) MCM-41 with eugenol. Scale markers of 100 nm.

Figure 1 shows the morphology of the here-obtained MCM-41 powder. Figure 1a,b present the SEM images of the MCM-41 powders with and without eugenol, respectively. One can observe that the silica particles presented a spherical shape with a mean size of around 100 nm, where the incorporation of eugenol slightly reduced their particle size. Therefore, the incorporation of eugenol did not alter the morphology of the mesoporous MCM-41 type nanoparticles. TEM was carried out in order to further ascertain the morphology of the MCM-41 particles. Figure 1c confirmed the spherical shape of the MCM-41 particle without eugenol, showing that their mean size was 96.1 ± 3.8 nm. A similar morphology can be observed in Figure 1d for the MCM-41 powder with eugenol, having a mean diameter of 88.6 ± 2.1 nm. Similar results were reported by Ribes et al. [40] in which the immobilization of eugenol and thymol on the surface of MCM-41 did not affect the integrity of the mesoporous silica particles. Also, Ruiz-Rico et al. [41] observed that the appearance of fumed silica, amorphous silica, and MCM-41 particles did not change after functionalization with thymol. Indeed, MCM-41 has been widely used as a model material in the context of porosity characterization owing to its peculiar features, such as high surface area, large pore volume, low toxicity, high chemical and thermal stability, and versatile chemical modifiable surface. It has been reported that the pore structure is organized in the form of hexagonal arrays of uniform tubular channels of controlled width [47,48]. As a result, mesoporous silica nanoparticles are excellent candidates for reference adsorbents for standardizing adsorption measurements and methods for characterization of porous solids due to their regular pore structure, high stability, and also convenient method of synthesis [49,50].

**Figure 2:**
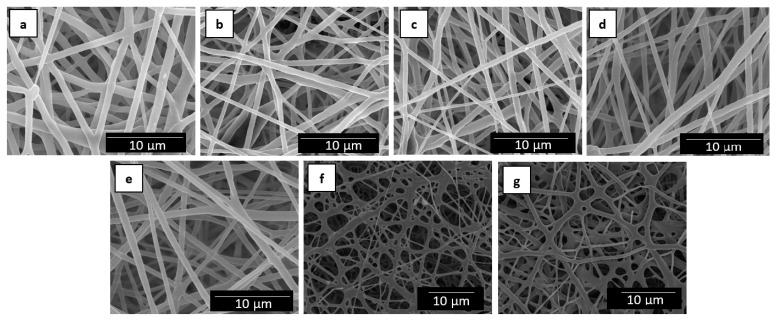
Scanning electron microscopy (SEM) images of the electrospun fibers of poly(3-hydroxybutyrate-*co*-3-hydroxyvalerate) (PHBV)/Mobil Composition of Matter (MCM)-41 with eugenol: (**a**) Neat PHBV; (**b**) 2.5 wt.-% MCM-41 + eugenol; (**c**) 5 wt.-% MCM-41 + eugenol; (**d**) 7.5 wt.-% MCM-41 + eugenol; (**e**) 10 wt.-% MCM-41 + eugenol; (**f**) 15 wt.-% MCM-41 + eugenol; (**g**) 20 wt.-% MCM-41 + eugenol. Scale markers of 10 µm.

Figure 2 shows the resultant electrospun mats obtained from the neat PHBV solution and the different solutions of PHBV/MCM-41 with eugenol. One can observe that, in all cases, the electrospinning process generated a mat composed of non-woven fibers with a similar morphology. Table 1 summarizes the mean diameters of the electrospun fibers. The neat PHBV fibers without MCM-41, processed in the same conditions, presented a mean diameter of 0.89 ± 0.30 µm. It can be observed that the mean diameters of the electrospun fibers varied in the 0.6–0.7 µm range when the silica particles were incorporated. However, one can observe that the electrospun fibers with the highest particle contents, that is, 15 and 20 wt.-% MCM-41, presented certain cross-linking or fibers coalescence. This can be related to difficulties encountered during the fiber formation more likely due to a phenomenon of particle aggregation in the electrospinning process. Indeed, it is known that high nano-sized filler contents habitually lead to the formation of beaded regions in the electrospun fibers [51,52].

**Figure 3:**
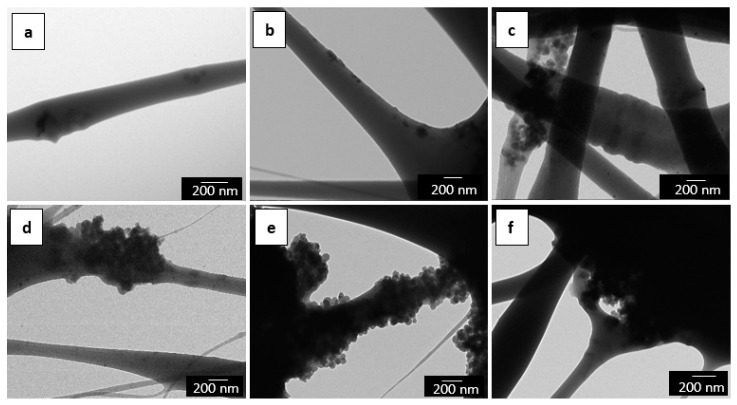
Transmission electron microscopy (TEM) images of the electrospun fibers of poly(3-hydroxybutyrate-*co*-3-hydroxyvalerate) (PHBV)/Mobil Composition of Matter (MCM)-41 with eugenol: (**a**) 2.5 wt.-% MCM-41 + eugenol; (**b**) 5 wt.-% MCM-41 + eugenol; (**c**) 7.5 wt.-% MCM-41 + eugenol; (**d**) 10 wt.-% MCM-41 + eugenol; (**e**) 15 wt.-% MCM-41 + eugenol; (**f**) 20 wt.-% MCM-41 + eugenol. Scale markers of 200 nm.

TEM was also performed in order to evaluate the distribution of the MCM-41 particles inside the electrospun fibers. The detailed morphologies of the electrospun mats of PHBV/MCM-41 with eugenol, at different particle contents, are shown in Figure 3. One can observe that at low contents, that is, from 2.5 wt.-% to 7.5 wt.-% MCM-41 with eugenol, the functionalized silica nanoparticles were relatively well distributed inside the electrospun fibers. However, for higher filler contents, the MCM-41 particles were mainly agglomerated in certain regions of the fibers. This fact supports the above-described morphology during the SEM analysis by which the silica nanoparticles interconnected the fibers in the electrospun mats. A similar morphology was recently reported, for instance, by Cherpinski et al. [53] in PHB fibers containing palladium nanoparticles (PdNPs).

**Figure 4:**
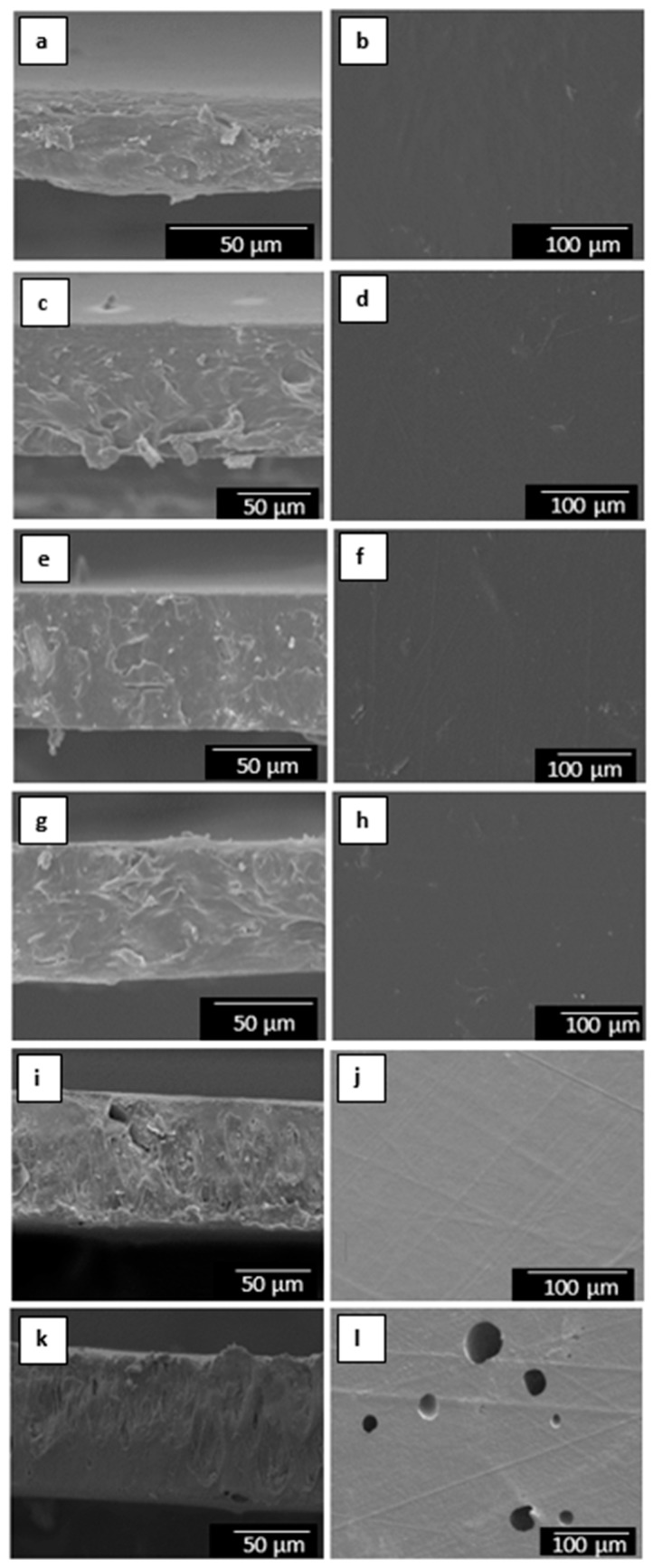
Scanning electron microscopy (SEM) images of the films cross-section (left) and top view (right) of poly(3-hydroxybutyrate-*co*-3-hydroxyvalerate) (PHBV)/Mobil Composition of Matter (MCM)-41 with eugenol: (**a**,**b**) 2.5 wt.-% MCM-41 + eugenol; (**c**,**d**) 5 wt.-% MCM-41 + eugenol; (**e**,**f**) 7.5 wt.-% MCM-41 + eugenol; (**g**,**h**) 10 wt.-% MCM-41 + eugenol; (**k**,**j**) 15 wt.-% MCM-41 + eugenol; (**k**,**l**) 20 wt.-% MCM-41 + eugenol. Scale markers of 50 µm and 100 µm.

The morphology of the electrospun materials was also analyzed by SEM in order to ascertain the effect of the film-forming process on the PHBV fibers. Figure 4 shows the SEM images at both the cross-section and surface of the electrospun PHBV materials containing different amounts of MCM-41 with eugenol. The surface cryo-fractures of the electrospun materials, shown in the left column, revealed the formation of a continuous film with much reduced porosity. This process has been ascribed to a process of fibers coalescence that occurs during annealing, that is, at a temperature below the polymer’s T_m_ [54]. In the case of the electrospun films having the highest particle contents, that is, 15 and 20 wt.-% MCM-41 with eugenol, the films presented a higher porosity and also certain plastic deformation. This observation can be related to the above-described fiber morphology and, more importantly, to the presence of high loadings of eugenol that could plasticize the PHBV matrix and/or migrate during the annealing process. In the top view of the electrospun films, shown in the right column, one can clearly observe that the film sample containing 20 wt.-% MCM-41 presented higher porosity on its surface. This morphology confirms that contents above 15 wt.-% MCM-41 with eugenol are not optimal to be processed by electrospinning and thermally post-treatment at 160 °C. Similar findings were concluded when electrospun mats of PHBV with ~20 mol.-% HV were post-treated at higher temperatures than optimal, resulting in an increased porosity due to partial polymer melting and/or degradation [55].

**Figure 5:**
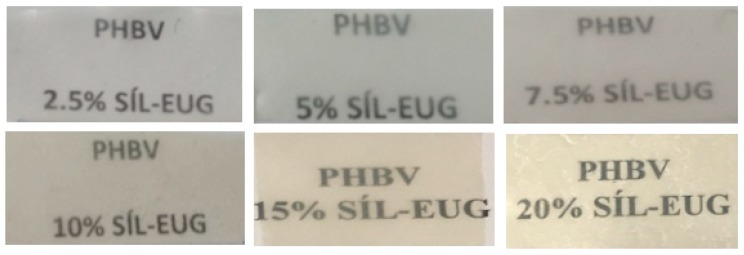
Visual aspect of the electrospun films of poly(3-hydroxybutyrate-*co*-3-hydroxyvalerate) (PHBV)/Mobil Composition of Matter (MCM)-41 with eugenol.

Figure 5 shows the visual aspect of the resulting annealed electrospun PHBV films containing MCM-41 with eugenol. Although the contact transparency of the films was similar in all the samples, the films with the highest particle contents, that is, 15 and 20 wt.-% MCM-41 with eugenol, developed a yellow color. A similar yellowing and, in some cases, browning was previously observed by Muratore et al. [56] after the incorporation of eugenol into commercial paper prepared by grafting of this EO onto cellulose at 120–180 °C. This effect was ascribed to the intrinsic eugenol color, which is a pale yellow oily liquid, as well as secondary reactions and/or by-products due to thermal oxidation and chain scission of the substrate favored by high temperatures and prolonged time. Therefore, the incorporation of up to 10 wt.-% MCM-41 with eugenol successfully allows the production of contact transparent films of PHBV.

### 3.2. Thermal Properties

Table 2 displays the main thermal transitions, obtained by DSC during the heating and cooling steps, of the annealed electrospun neat PHBV film and the films containing MCM-41 without and with eugenol. It can be observed that the neat PHBV film presented a glass transition temperature (T_g_) of 2.6 ± 0.4, while the addition of MCM-41 without eugenol had a negligible effect on T_g_. Interestingly, after the incorporation of MCM-41 with eugenol, the T_g_ values were reduced to 1.8–0.6 °C in the PHBV film samples. Reductions of T_g_ are habitually associated to a plasticization process by low-molecular weight (M_W_) molecules with high chemical affinity to the polymer matrix by which the free volume of the polymer is enlarged since they increase the distance between the polymer chains and then favor segmental motion [57]. Some previous studies have already reported the plasticizing effect of eugenol on different polymer matrices. For instance, Fernandes Nassar et al. [58] reported a reduction in T_g_ when eugenol was incorporated into soy protein isolate (SPI) films, ascribing this effect to the plasticizing role that the aroma compound played in the protein matrix. Also, Narayanan et al. [59] observed a reduction in T_g_ from 4 °C, for the neat PHB film, to −14 °C, for PHB films containing up to 200 μg/g of eugenol.

Whereas cold crystallization phenomenon was not observed in any of the PHBV films during heating, all the samples crystallized from the melt during cooling. In particular, the neat PHBV film showed a crystallization temperature (T_c_) of 116.8 ± 0.5 °C. The presence of MCM-41 without eugenol increased the crystallization temperature of PHBV, up to reaching a maximum value of 120.5 °C for the film filled at 15 wt.-%. This result suggests that the nanoparticles provided a nucleating effect on the PHBV molecules, except for the film filled with 20 wt.-% MCM-41, possibly due to nanoparticles agglomeration as previously described during the morphological analysis. On the contrary, one can observe that the T_c_ values of the film samples containing MCM-41 with eugenol decreased as the filler content increased. Then, the T_c_ value was reduced up to a value of 113.8 ± 0.6 °C for the film filled with 20 wt.-% MCM-41 with eugenol. This restrained crystallization of PHBV can be ascribed to the above-described plasticizing effect of eugenol, which impair the packing of the polymer chains to form crystals.

During heating, the neat PHBV film melted in a single peak at 170.4 ± 0.2 °C while all the PHBV films containing MCM-41 without eugenol presented similar T_m_ values in the 169–171 °C range. However, the T_m_ values progressively reduced in the PHBV films containing MCM-41 with eugenol was as the filler content increased. Up to contents of 15 wt.-% MCM-41 with eugenol, the PHBV films presented a single melting peak in the 163–171 °C range, whereas the film filled with 20 wt.-% MCM-41 with eugenol showed two endothermic peaks, starting melting at 160.5 ± 1.5 °C. Therefore, the MCM-41 particles when loaded with eugenol were able to impair and induce some defects in the PHBV crystals, particularly at the highest tested contents. It is also worthy to note that the presence of MCM-41 without eugenol, up to fillings of 15 wt.-%, increased the values of enthalpy of melting (ΔH_m_), confirming the formation of more perfect PHBV crystals with thicker lamellae by a nucleation phenomenon. As opposite, all the PHBV films with MCM-41 with eugenol presented lower values of ΔH_m_, being this reduction significantly noticeable for the films filled with contents above 15 wt.-%. Therefore, the presence of MCM-41 with eugenol impaired the crystallization of PHBV due to the above-described reduction of the biopolymer segments packing. It has been similarly reported that the addition of mesoporous silica nanoparticles has a slight influence on T_g_ or T_m_ in polymer nanocomposites [60,61], therefore supporting that the here-observed suppressed effect on the melt behavior is ascribed to eugenol. In this sense, Garrido-Miranda et al. [62] showed that the T_m_ value of PHB/thermoplastic starch (TPS)/organically modified montmorillonite (OMMT) nanocomposites was reduced by approximately 4 °C when 3 wt.-% eugenol was incorporated, concluding that eugenol induces the formation of less perfect crystals. Woranuch et al. [63] also observed a ΔH_m_ reduction when eugenol-loaded chitosan nanoparticles were incorporated into thermoplastic flour (TPF) made of cassava, rice, and waxy rice through an extrusion process. The reduction observed was related to a plasticization by eugenol.

**Figure 6:**
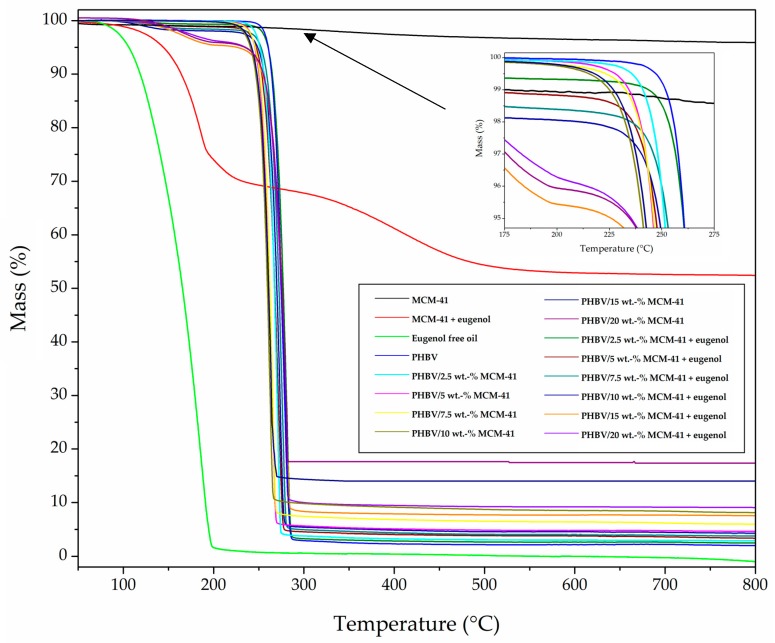
Thermogravimetric analysis (TGA) curves for Mobil Composition of Matter (MCM)-41, eugenol, MCM-41 with eugenol, poly(3-hydroxybutyrate-*co*-3-hydroxyvalerate) (PHBV), and PHBV/Mobil Composition of Matter (MCM)-41 without and with eugenol.

Figure 6 depicts the TGA curves of MCM-41 and MCM-41 with eugenol powders, the eugenol-free oil, and the electrospun films made of neat PHBV and PHBV/MCM-41 without and with eugenol. Table 3 gathers the main relevant thermal parameters obtained from the TGA curves. As one can observe in the graph, the neat MCM-41 particles presented a mass loss of ~5% at a temperature close to 100 °C, which can be ascribed to residual humidity on the surface and/or in the pores of the nanoparticles. In addition, the neat MCM-41 particles provided a residual mass of 95.0 ± 2.3% measured at 800 °C. On the contrary, the eugenol free oil had a relatively low thermal stability, showing full decomposition at approximately 200 °C. Moreover, comparison of the TGA curves of the MCM-41 nanoparticles with and without eugenol corroborated that the eugenol loading was 49.5 ± 1.2%. This loading capacity of MCM-41 was higher than other encapsulation techniques reported for polyphenols [64].

In relation to the neat PHBV film, a low-intense first weight loss process (<1%) was observed at 100 °C due to absorbed moisture and/or volatiles leaving the samples. Trapped solvent losses were discarded by Fourier transform infrared (FTIR) spectroscopy and TGA of the neat PHBV fibers (results not shown). One can also observe that the biopolymer presented the onset of degradation, measured at the temperature at which the mass loss was 5% (T_5%_), at 259.9 ± 1.2 °C. The degradation temperature (T_deg_) occurred at 277.3 ± 0.6 °C, degrading in a single step and producing a residual mass of 2.0 ± 0.2% at 800 °C. In addition, the weight loss process corresponding to thermal decomposition reaction of the biopolymer chain occurred sharply, approximately from 225 °C to 275 °C. The thermal degradation onset was shifted to lower temperatures when both the MCM-41 without and with eugenol, in all the composition range, was incorporated. This result suggests that the nanoparticles catalyzed thermal degradation. Interestingly, the T_5%_ and T_deg_ values were slightly improved at the lowest content of MCM-41 with eugenol, which can be related to the above-described nucleating effect and restricted mobility of the biopolymer chains by the presence of MCM-41 and eugenol. However, the thermal stability was reduced at the higher filler contents, that is, 15 wt.-% and 20 wt.-% MCM-41 with eugenol, due to the high content of both MCM-41 and eugenol. Furthermore, the residual weight at 800 °C of the PHBV/MCM-41 with eugenol films increased due to the presence of the mesoporous silica nanoparticles. In any case, the incorporation of up to 10 wt.-% of MCM-41 with eugenol had a relatively low influence on the thermal stability of the PHBV films, which can be considered a positive result since they encapsulate an active component with low thermal stability. In this sense, Requena et al. [65] reported that the incorporation of carvacrol and eugenol enhanced the thermal sensitivity of PHBV, decreasing the onset temperature, whereas the incorporation of whole essential oils (oregano and clove) slightly promoted its thermal stability. The latter effect was suggested to occur due to a strong bonding of the eugenol with the polymer network.

### 3.3. Mechanical Properties

Since the resultant electrospun films may be subjected to various kinds of stress during use, the determination of the mechanical properties involves not only scientific but also technological and practical aspects. Table 4 displays the values of elastic modulus (E), tensile strength at yield (σ_y_), elongation at break (ε_b_), and toughness (T) of the electrospun films made of PHBV and PHBV/MCM-41 with eugenol calculated from their strain–stress curves. In general, all the electrospun films presented characteristics of a brittle material associated to the inherent low ductility of PHBV, showing ε_b_ and T values below 3% and 0.5 mJ/m^3^, respectively. The film specimens also presented a relative high mechanical strength. In particular, the mean values of E were comprised in the of 1250–2000 MPa range while σ_y_ varied from approximately 18 to 30 MPa. The here-obtained mechanical properties of the PHBV films are similar to those recently reported in our group by Cherpinski et al. [54] for PHB films also prepared by electrospinning and thereafter thermally post-treated, having a E value of 1104 MPa and ε_b_ and T values of 2.9% and 0.3 mJ/m^3^, respectively.

It can be observed that the incorporation of MCM-41 with eugenol increased the mechanical strength of the PHBV films while the ductility was slightly reduced. This effect can be related to the reinforcing effect of MCM-41 as a filler in the PHBV matrix, while the smaller impact in ductility may be accounted for the plasticizing effect that the released eugenol may have in the polymer matrix. This mechanical enhancement of E and σ_y_ indicates a good transfer of mechanical energy from the hard filler, that is, MCM-41, as well as the interaction between the biopolymer matrix and the silica nanoparticles. Considering both the low concentration of MCM-41 and the presence of eugenol, which acts as plasticizer, the mechanical reinforcement of the filler is thought to dominate the enhancement in E and σ_y_. However, a comparative reduction, change in trend, in mechanical strength was observed when the content of the antimicrobial filler exceeded 10 wt.-%. This effect may be ascribed to a balance between filler agglomeration and stronger plasticizing effect of the released eugenol. High tensile strengths are generally necessary for food packaging films in order to withstand the normal stress encountered during their application, subsequent shipping, and handling [66]. Similarly, Voon et al. [67] reported that the addition of 3 wt.-% of mesoporous silica nanoparticles to bovine gelatin films improved their mechanical resistant properties, that is, σ_y_, while it reduced ε_b_. Others studies have also demonstrated that the incorporation of mesoporous silica nanoparticles can remarkably enhance the mechanical strength in PVOH-based materials due to the intermolecular interactions between the fillers and the polymer when prepared by in situ radical copolymerization [68,69]. As compared to commercial biopolymers for packaging applications, the here-developed electrospun films of PHBV/MCM-41 with eugenol are slightly less deformable but more elastic than thermo-compressed PHBV films, stiffer but less ductile than rigid polylactide (PLA) films, and mechanically stronger but considerably more brittle than flexible poly(butylene adipate-*co*-terephthalate) (PBAT) [70].

### 3.4. Barrier properties

Table 5 gathers the WVP and LP values of the electrospun PHBV/MCM-41 with eugenol films. It can be observed that the incorporation of low contents of MCM-41 with eugenol, that is, 2.5 and 5 wt.-%, induced an increase in the WVP values of the electrospun PHBV films while the water vapor barrier properties were improved for contents higher than 7.5 wt.-%. A similar effect was observed in the case of LP. The resultant increase in permeability observed at low filler loadings can be related to the plasticizing effect of eugenol on the PHBV matrix outweighing the barrier effect of the MCM-41 filler, as above discussed during the thermal analysis, with a subsequent increase in the matrix free volume. At higher contents, however, the barrier improvements can be ascribed to the presence of large quantities of mesoporous silica nanoparticles. Then, MCM-41 successfully acted as barrier elements forcing the permeant molecules to travel through a longer path to permeate across according to the early theory suggested by Nielsen [71]. It is also worthy to mention the change in the barrier trend observed for the film samples containing the highest filler contents, that is, 20 wt.-% MCM-41 with eugenol. This permeability change in trend can also be ascribed to the above-mentioned higher filler agglomeration resulting in a somewhat increased porosity as observed in the morphological analysis, leading to preferential paths for diffusion. 

The here-prepared electrospun films of PHBV/MCM-41 with eugenol showed higher WVP values than PHBV films with 12 mol.-% HV prepared by solvent casting, that is, 1.27 × 10^−14^ kg·m·m^−2^·Pa^−1^·s^−1^ [72] or PHB films obtained by compression-molded, that is, 1.7 × 10^−15^ kg·m·m^−2^·Pa^−1^·s^−1^ [73], which can be related to the higher mol.-% HV fraction in the here-used copolyester. Therefore, it can be considered that intermediate contents of MCM-41 with eugenol inside the fibers promoted lower free volume available for diffusion. Similar results were reported for instance by Hashemi Tabatabaei et al. [74], who showed that the incorporation of 5 wt.-% mesoporous silica microparticles decreased the WVP value from 8.9 g·m·m^−2^·Pa^−1^·s^−1^ to 1.6 × 10^−11^ g·m·m^−2^·Pa^−1^·s^−1^ in gelatin/k-carrageenan films. Also, Hassannia-Kolaee et al. [75] reported a reduction of up to approximately 33% in WVP for whey protein isolate (WPI)/pullulan (PUL) films containing 1, 3, and 5 wt.-% mesoporous silica nanoparticles prepared by a casting method. The barrier improvement achieved was attributed to the formation of hydrogen bonds between the polymer hydroxyl groups and the oxygen atoms of silica and also to the good dispersion of the nanoparticles in the polymer matrix. In relation to eugenol, some studies have also demonstrated that the direct incorporation of EOs, among them eugenol, in polymer films do not induce significant change in WVP, concluding that water permeability basically depends on the hydrophilic–hydrophobic ratio of the film constituents [76,77]. Other studies have reported that the addition of EOs can negatively increase the water permeability depending on the nature of the polymer matrix and the type and concentration of EO [78]. However, this impairment may be attributed to the difficulties encountered to integrate the hydrophobic EO in hydrophilic networks that might cause matrix disruptions and create void spaces at the polymer–oil interface [79]. However, in the current study, the expected plasticizing effect of the hydrophobic eugenol within the PHBV matrix is seen detrimental for the barrier performance at lower silica loadings. 

### 3.5. Antimicrobial activity

*S. aureus* and *E. coli* are common microorganisms associated with food-related diseases. Therefore, the incorporation of active substances in the design of packaging materials can be an important technology not only to avoid food waste but also to enhance food safety [80]. For the pure eugenol in its original liquid form, the MIC and BIC values for *S. aureus* were 1.25 µL/mL and 2.5 µL/mL, respectively, and for *E. coli* these values were 2.5 µL/mL and 5 µL/mL, respectively. The MCM-41 particles with eugenol presented a MIC value against *S. aureus* and *E. coli* of 10 µg/mL and 20 µg/mL, respectively, while the BIC values were 40 µg/mL for both bacteria. The higher value observed for *E. coli* can be ascribed to the greater bacterial resistance of G- bacteria than G+ ones [81], thus a higher dose of the antimicrobial was needed to obtain the same efficacy.

The antimicrobial activity of the film samples was evaluated using the JIS Z2801. The reduction values in the open system against *S. aureus* and *E. coli* are gathered in Table 6 and Table 7, respectively. Table 8 and Table 9 includes the values against *S. aureus* and *E. coli*, respectively, in the closed system. As expected, it can be observed that both the unfilled PHBV film and the different PHBV films containing MCM-41 without eugenol showed no inhibition effect on the bacterial growth (R ≤ 1). In contrast, the incorporation of MCM-41 with eugenol into the PHVB film exhibited significant antibacterial activity against both bacteria. In the open system, at the initial day, that is, for the tests carried out the same day of the film production, the bacterial reduction on the film surface gradually increased with the content of MCM-41 with eugenol. As it can be seen in Table 6 for *S. aureus*, at the lowest contents, that is, 2.5 and 5 wt.-% MCM-41 with eugenol, the films presented a slight antibacterial activity (R ≥ 1 and < 2). For the highest tested contents, that is, 7.5 and 10 wt.-% MCM-41 with eugenol, the films generated a significant surface reduction (R ≥ 1 and < 3). Although none of the films produced a strong reduction (R ≥ 3), materials with values of surface reduction in the 1–2 range are usually considered as bacteriostatic [82]. Therefore, electrospun PHBV films with 10 wt.-% MCM-41 with eugenol were able to provide a bacteriostatic effect against *S. aureus*. As also shown in the table, after 15 days, the films still kept a significant antibacterial activity. In particular, the films with 7.5 and 10 wt.-% MCM-41 with eugenol still presented significant values of reduction (R ≥ 1 and < 3) while these presented slight values (R ≥ 0.5 and < 1) for loadings of 2.5 and 5 wt.-%. This suggests that, although part of eugenol was released from the films, MCM-41 was still able to retain over time a significant amount of EO. Regarding *E. coli*, shown in Table 7, the required concentration of MCM-41 with eugenol to generate an antimicrobial effect in the open system was 15 wt.-%. At this content, the films presented a significant value of reduction, that is, R values of 1.30 and 1.40 at days 0 and 15, respectively. This supports the above-described higher antimicrobial resistance of *E. coli*, as a G- bacterium, which would need more exposure time to the active oil to render a similar antimicrobial activity.

The tested closed system was aimed to better represent the real conditions in a packaging material. In the case of *S. aureus*, which is shown in Table 8, the film with 10 wt.-% MCM-41 with eugenol was selected since this sample showed a high R value at a relatively low content of filler. One can observe that the antimicrobial activity was higher than that observed in the open system, showing R values of 1.35 and 1.64 for day 0 and 15, respectively. This confirms the high volatility of eugenol, which remained enclosed and still active in the system in comparison to the open one. In Table 9, for *E. coli*, the R values were 1.34 and 1.58 for day 0 and 15, respectively, in the closed system. Therefore, the here-achieved antimicrobial effect was somehow higher in the closed system than in the open one. This result has been recently ascribed to the volatile portion of active components accumulated in the system’s headspace, which successfully contributed to decrease bacterial growth [83]. In any case, the differences in bacterial reduction in both tested packaging conditions, that is, the open and closed systems, for each type of bacteria was relatively low. This observation can be related to the use of MCM-41 that successfully performed as vehicles to control the release of eugenol and to render high antimicrobial activity.

Similar to this study, other authors have previously reported the antibacterial activity of eugenol in different biopolymer articles. For instance, PCL/gelatin electrospun membranes loaded with active peptide containing 30 wt.-% of eugenol successfully inhibited the growth of *E. coli* and *S. aureus* with inhibition rates of 71.6% and 78.6%, respectively [84]. In another study, compression-molded PHBV bilayer films were sprayed with four active components, among them eugenol, resulting in antimicrobial systems against G- and G+ bacteria such as *E. coli* and *Listeria innocua* (*L. innocua*) [65]. In this previous research, the added active agents were more effective against G- than G+, which in agreement with the present results. The benefit of loading antimicrobial agents in MCM-41 has been also studied elsewhere, both against bacteria and fungi. For instance, Park et al. [85] loaded allyl isothiocyanate, a natural antimicrobial, in MCM-41 as a novel controlled release vector against selected foodborne pathogenic microorganisms. In other studies, other volatile EOs were immobilized on the surface of mesoporous silica materials acting as antifungal agents and showing improved antimicrobial activity than the free compounds [39,40]. 

## 4. Conclusions

EOs are well known for their antimicrobial properties, being suitable as food preservatives. However, to ensure their long-term effect, which is controlled by their volatility, it may be necessary to encapsulate them in, for instance, porous materials. The present study evaluated the complexation of eugenol EO on MCM-41 to be thereafter incorporated into PHBV biopolymers by electrospinning. The resultant electrospun mats were annealed below the biopolymer melting point to generate continuous films. The thermal analysis performed on the films showed that the incorporation of MCM-41 with eugenol induced certain plasticization on PHBV as well as a reduction in crystallinity. Interestingly, the incorporation of MCM-41 with eugenol up to 10 wt.-% had a relatively low influence on the thermal stability of the PHBV films. During the mechanical analysis, it was observed that the mechanical strength of the PHBV films was increased while the ductility was only slightly reduced after the incorporation of MCM-41 with eugenol. The barrier properties were also enhanced due to the presence of the eugenol-containing nanofillers and were optimal around contents of 15 wt.-%. Finally, the antimicrobial activity against *S. aureus* and *E. coli* was studied in both an open and closed system to better represent the real conditions in packaging applications. The electrospun biopolymer films showed antibacterial activity after 15 days, being higher (as expected) in the ones that were studied in the closed system, which was ascribed to the accumulation of eugenol in the system’s headspace. For all this, the films developed can be regarded as a sustainable material to be used in the form of interlayers or coatings for active food packaging applications.

## Figures and Tables

**Table 1:** Mean diameters of the electrospun fibers of poly(3-hydroxybutyrate-*co*-3-hydroxyvalerate) (PHBV)/Mobil Composition of Matter (MCM)-41 with eugenol.

Fibers	Diameter (µm)
PHBV	0.89 ± 0.30
PHBV/2.5 wt.-% MCM-41 + eugenol	0.65 ± 0.19
PHBV/5 wt.-% MCM-41 + eugenol	0.66 ± 0.16
PHBV/7.5 wt.-% MCM-41 + eugenol	0.63 ± 0.18
PHBV/10 wt.-% MCM-41 + eugenol	0.64 ± 0.19
PHBV/15 wt.-% MCM-41 + eugenol	0.65 ± 0.19
PHBV/20 wt.-% MCM-41 + eugenol	0.67 ± 0.24

**Table 2:** Thermal properties in terms of glass transition temperature (T_g_), crystallization temperature (T_c_), melting temperature (T_m_), and normalized enthalpy of melting (ΔH_m_) for the electrospun films of poly(3-hydroxybutyrate-*co*-3-hydroxyvalerate) (PHBV) and PHBV/Mobil Composition of Matter (MCM)-41 without and with eugenol.

Film	T_g_ (°C)	T_c_ (°C)	T_m_ (°C)	ΔH_m_ (J/g)
PHBV	2.6 ± 0.4	116.8 ± 0.5	170.4 ± 0.2	83.2 ± 3.0
PHBV/2.5 wt.-% MCM-41	2.6 ± 0.2	117.8 ± 0.6	169.9 ± 0.1	85.7 ± 0.5
PHBV/5 wt.-% MCM-41	2.3 ± 0.5	118.1 ± 0.4	170.1 ± 0.6	86.2 ± 4.0
PHBV/7.5 wt.-% MCM-41	2.4 ± 0.4	118.2 ± 0.2	171.7 ± 2.1	87.4 ± 7.3
PHBV/10 wt.-% MCM-41	2.3 ± 0.3	118.9 ± 0.1	170.2 ± 0.6	89.7 ± 6.3
PHBV/15 wt.-% MCM-41	2.2 ± 0.2	120.5 ± 0.3	170.1 ± 1.0	100.6 ± 7.4
PHBV/20 wt.-% MCM-41	2.5 ± 0.1	116.9 ± 0.1	169.0 ± 0.1	67.9 ± 5.9
PHBV/2.5 wt.-% MCM-41 + eugenol	1.8 ± 0.6	116.7 ± 0.1	169.0 ± 1.6	77.4 ± 3.4
PHBV/5 wt.-% MCM-41 + eugenol	1.4 ± 0.8	116.4 ± 0.6	167.4 ± 0.3	74.9 ± 6.2
PHBV/7.5 wt.-% MCM-41 + eugenol	1.5 ± 0.3	118.5 ± 0.3	168.8 ± 3.6	72.3 ± 6.9
PHBV/10 wt.-% MCM-41 + eugenol	1.3 ± 0.4	114.9 ± 0.7	165.4 ± 0.4	66.8 ± 4.8
PHBV/15 wt.-% MCM-41 + eugenol	0.9 ± 0.5	113.9 ± 0.2	163.4 ± 0.3	50.3 ± 4.5
PHBV/20 wt.-% MCM-41 + eugenol	0.6 ± 0.2	113.8 ± 0.6	160.5 ± 1.5/168.6 ± 0.1	47.4 ± 2.1

**Table 3:** Thermal properties in terms of mass loss was 5% (T_5%_), degradation temperature (T_deg_), mass loss at T_deg_, and a residual mass at 800 °C for Mobil Composition of Matter (MCM)-41, MCM-41 with eugenol, eugenol free oil, and electrospun films of poly(3-hydroxybutyrate-*co*-3-hydroxyvalerate) (PHBV) and PHBV/MCM-41 without and with eugenol.

Sample	T_5%_ (°C)	T_deg_ (°C)	Mass Loss (%)	Residual Mass (%)
MCM-41 powder	-	-	-	95.0 ± 2.3
MCM-41 with eugenol powder	143.7 ± 2.4	178.3 ± 0.7	16.3 ± 0.5	52.4 ± 1.9
Eugenol free oil	105.9 ± 3.2	185.5 ± 0.6	80.6 ± 1.1	0.9 ± 0.1
PHBV	259.9 ± 1.2	277.3 ± 0.6	62.0 ± 0.8	2.0 ± 0.2
PHBV/2.5 wt.-% MCM-41	250.8 ± 2.3	270.9 ± 0.2	74.4 ± 1.2	3.3 ± 0.4
PHBV/5 wt.-% MCM-41	245.3 ± 2.7	265.4 ± 1.0	72.1 ± 0.6	5.4 ± 0.8
PHBV/7.5 wt.-% MCM-41	245.3 ± 2.2	265.4 ± 0.8	70.3 ± 0.3	8.0 ± 0.4
PHBV/10 wt.-% MCM-41	240.7 ± 1.8	262.7 ± 0.5	70.8 ± 0.2	9.1 ± 1.0
PHBV/15 wt.-% MCM-41	230.0 ± 2.0	262.0 ± 0.2	70.2 ± 0.7	14.6 ± 0.9
PHBV/20 wt.-% MCM-41	215.2 ± 1.7	256.4 ± 0.6	80.5 ± 0.9	17.2 ± 0.2
PHBV/2.5 wt.-% MCM-41 + eugenol	259.8 ± 2.6	281.0 ± 2.5	71.3 ± 0.3	2.5 ± 0.4
PHBV/5 wt.-% MCM-41 + eugenol	251.7 ± 1.4	276.4 ± 1.6	71.4 ± 0.3	3.4 ± 0.7
PHBV/7.5 wt.-% MCM-41 + eugenol	248.0 ± 2.7	273.7 ± 0.9	72.8 ± 0.4	3.8 ± 0.8
PHBV/10 wt.-% MCM-41 + eugenol	247.1 ± 3.2	271.8 ± 0.7	73.9 ± 0.7	4.3 ± 1.0
PHBV/15 wt.-% MCM-41 + eugenol	215.2 ± 7.3	261.0 ± 4.2	74.6 ± 1.4	7.5 ± 3.6
PHBV/20 wt.-% MCM-41 + eugenol	205.1 ± 5.1	259.2 ± 4.4	75.6 ± 1.2	9.1 ± 3.7

**Table 4:** Mechanical properties in terms of elastic modulus (E), tensile strength at yield (σ_y_), elongation at break (ε_b_), and toughness (T) for the electrospun films of poly(3-hydroxybutyrate-*co*-3-hydroxyvalerate) (PHBV) and PHBV/Mobil Composition of Matter (MCM)-41 with eugenol.

Film	E	$\sigma_{y}$ (MPa)	$\varepsilon_{b}$ (%)	T (mJ/m^3^)
PHBV	1252 ± 79	18.1 ± 2.1	2.4 ± 0.3	0.3 ± 0.1
PHBV/2.5 wt.-% MCM-41 + eugenol	1735 ± 60	27.0 ± 2.4	2.4 ± 0.1	0.4 ± 0.1
PHBV/5 wt.-% MCM-41 + eugenol	1976 ± 162	25.1 ± 7.8	1.7 ± 0.4	0.2 ± 0.1
PHBV/7.5 wt.-% MCM-41 + eugenol	2000 ± 365	27.7 ± 5.4	2.2 ± 0.2	0.4 ± 0.1
PHBV/10 wt.-% MCM-41 + eugenol	1802 ± 288	21.7 ± 5.8	1.8 ± 0.2	0.2 ± 0.1
PHBV/15 wt.-% MCM-41 + eugenol	1702 ± 140	29.4 ± 3.4	2.0 ± 0.3	0.3 ± 0.1
PHBV/20 wt.-% MCM-41 + eugenol	1462 ± 358	25.1 ± 5.4	2.1 ± 0.5	0.3 ± 0.2

**Table 5:** Permeability values in terms of water vapor permeability (WVP) and d-limonene permeability (LP) for the electrospun films of poly(3-hydroxybutyrate-*co*-3-hydroxyvalerate) (PHBV) and PHBV/Mobil Composition of Matter (MCM)-41 with eugenol.

Sample	WVP × 10^−14^ (kg·m·m^−2^·Pa^−1^·s^−1^)	LP × 10^−14^ (kg·m·m^−2^·Pa^−1^·s^−1^)
PHBV	5.34 ± 1.79	2.68 ± 1.82
PHBV/2.5 wt.-% MCM-41 + eugenol	8.68 ± 3.57	3.41 ± 0.97
PHBV/5 wt.-% MCM-41 + eugenol	8.84 ± 4.36	3.49 ± 1.17
PHBV/7.5 wt.-% MCM-41 + eugenol	4.25 ± 4.04	3.51 ± 0.54
PHBV/10 wt.-% MCM-41 + eugenol	2.99 ± 0.95	2.32 ± 0.68
PHBV/15 wt.-% MCM-41 + eugenol	0.25 ± 0.19	0.38 ± 0.20
PHBV/20 wt.-% MCM-41 + eugenol	4.08 ± 1.98	4.66 ± 2.91

**Table 6:** Antibacterial activity against *Staphylococcus aureus* (*S. aureus*) in the open system for the electrospun films of poly(3-hydroxybutyrate-*co*-3-hydroxyvalerate) (PHBV) and PHBV/Mobil Composition of Matter (MCM)-41 with eugenol.

Films	Initial		After 15 Days	
	Bacterial Counts [log (CFU/mL)]	R	Bacterial Counts [log (CFU/mL)]	R
Control day 0	5.75 ± 0.09	-	5.75 ± 0.09	-
Control 24 h	5.67 ± 0.07	-	5.68 ± 0.03	-
PHBV	5.39 ± 0.56	0.28 ± 0.52	5.29 ± 0.41	0.38 ± 0.38
PHBV/2.5 wt.-% MCM-41	4.86 ± 0.54	0.81 ± 0.58	5.06 ± 0.48	0.61 ± 0.46
PHBV/2.5 wt.-% MCM-41 + eugenol	4.33 ± 0.35	1.04 ± 0.39	4.75 ± 0.09	0.92 ± 0.12
PHBV/5 wt.-% MCM-41	5.47 ± 0.58	0.20 ± 0.65	5.51 ± 0.09	0.16 ± 0.06
PHBV/5 wt.-% MCM-41 + eugenol	4.60 ± 0.23	1.07 ± 0.23	4.69 ± 0.14	0.99 ± 0.14
PHBV/7.5 wt.-% MCM-41	5.77 ± 0.07	0.10 ± 0.01	5.44 ± 0.55	0.24 ± 0.57
PHBV/7.5 wt.-% MCM-41 + eugenol	4.55 ± 0.06	1.12 ± 0.11	4.55 ± 0.12	1.12 ± 0.15
PHBV/10 wt.-% MCM-41	5.98 ± 0.57	0.31 ± 0.06	4.70 ± 0.06	0.97 ± 0.09
PHBV/10 wt.-% MCM-41 + eugenol	4.43 ± 0.24	1.23 ± 0.20	4.55 ± 0.07	1.24 ± 0.10

**Table 7:** Antibacterial activity against *Escherichia coli* (*E. coli*) in the open system for the electrospun films of poly(3-hydroxybutyrate-*co*-3-hydroxyvalerate) (PHBV) and PHBV/Mobil Composition of Matter (MCM)-41 with eugenol.

Films	Initial		After 15 Days	
	Bacterial Counts [log (CFU/mL)]	R	Bacterial Counts [log (CFU/mL)]	R
Control day 0	5.76 ± 0.01	-	5.71 ± 0.02	-
Control 24 h	6.81 ± 0.01	-	6.80 ± 0.02	-
PHBV	5.99 ± 0.07	0.82 ± 0.01	6.08 ± 0.03	0.72 ± 0.05
PHBV/15 wt.-% MCM-41	6.41 ± 0.01	0.40 ± 0.03	6.15 ± 0.04	0.65 ± 0.06
PHBV/15 wt.-% MCM-41 + eugenol	5.51 ± 0.02	1.30 ± 0.02	5.40 ± 0.01	1.40 ± 0.06

**Table 8:** Antibacterial activity against *Staphylococcus aureus* (*S. aureus*) in the closed system for the electrospun films of poly(3-hydroxybutyrate-*co*-3-hydroxyvalerate) (PHBV) and PHBV/Mobil Composition of Matter (MCM)-41 with eugenol.

Films	Initial		After 15 Days	
	Bacterial Counts [log (CFU/mL)]	R	Bacterial Counts [log (CFU/mL)]	R
Control day 0	5.61 ± 0.03	-	5.65 ± 0.01	-
Control 24 h	6.82 ± 0.06	-	6.85 ± 0.01	-
PHBV	6.23 ± 0.08	0.59 ± 0.01	6.11 ± 0.03	0.74 ± 0.05
PHBV/10 wt.-% MCM-41	6.30 ± 0.01	0.52 ± 0.03	6.09 ± 0.04	0.76 ± 0.06
PHBV/10 wt.-% MCM-41 + eugenol	5.47 ± 0.01	1.35 ± 0.15	5.21 ± 0.01	1.64 ± 0.09

**Table 9:** Antibacterial activity against *Escherichia coli* (*E. coli*) in the closed system for the electrospun films of poly(3-hydroxybutyrate-*co*-3-hydroxyvalerate) (PHBV) and PHBV/Mobil Composition of Matter (MCM)-41 with eugenol.

Films	Initial		After 15 Days	
	Bacterial Counts [log (CFU/mL)]	R	Bacterial Counts [log (CFU/mL)]	R
Control day 0	5.68 ± 0.03	-	5.66 ± 0.06	-
Control 24 h	6.83 ± 0.01	-	6.60 ± 0.01	-
PHBV	6.10 ± 0.01	0.73 ± 0.01	6.11 ± 0.03	0.49 ± 0.04
PHBV/15 wt.-% MCM-41	6.24 ± 0.01	0.59 ± 0.03	6.26 ± 0.06	0.34 ± 0.01
PHBV/15 wt.-% MCM-41 + eugenol	5.49 ± 0.03	1.34 ± 0.03	5.02 ± 0.07	1.58 ± 0.01
